# Body Mass Index and Associated Clinical Variables in Patients with Non-Celiac Wheat Sensitivity

**DOI:** 10.3390/nu11061220

**Published:** 2019-05-29

**Authors:** Pasquale Mansueto, Maurizio Soresi, Francesco La Blasca, Francesca Fayer, Alberto D’Alcamo, Antonio Carroccio

**Affiliations:** 1 Department of Health Promotion Sciences, Maternal and Infant Care, Internal Medicine and Medical Specialties (PROMISE), University of Palermo, 90129 Palermo, Italy; pasquale.mansueto@unipa.it (P.M.); maurizio.soresi@unipa.it (M.S.); francescolablasca@gmail.com (F.L.B.); francesca.fayer@libero.it (F.F.); adalcamo@hotmail.it (A.D.); 2 Department of Health Promotion Sciences, Maternal and Infant Care, Internal Medicine and Medical Specialties (PROMISE), University of Palermo, and Internal Medicine, Giovanni Paolo II Hospital, 92019 Sciacca (ASP Agrigento), Italy

**Keywords:** non-celiac wheat sensitivity (NCWS), Body Mass Index (BMI), autoimmune diseases, HLA haplotype, Celiac Disease (CD), Irritable Bowel Syndrome (IBS)

## Abstract

Background: Non-Celiac Wheat Sensitivity (NCWS) is still a largely undefined condition, due to the lack of a diagnostic marker. Few data are available about the nutritional characteristics of NCWS patients at diagnosis. Aims: To evaluate the proportion of NCWS patients who were underweight, normal weight, overweight, or obese at diagnosis, and to search for possible correlations between their Body Mass Index (BMI) and other NCWS-related disease characteristics. Patients and Methods: The clinical charts of 145 NCWS patients (125 F, 20 M, mean age 37.1 ± 11.4 years), diagnosed between January 2012 and March 2018, were reviewed. As a comparison, 84 celiac disease (CD) patients (73 F, 11 M, mean age 39.8 ± 13.9 years) were evaluated. All NCWS diagnoses were based on a double-blind placebo-controlled wheat challenge (DBPCWC) method. Results: BMI distribution was similar in the NCWS (6.2% underweight and 15.2% obese subjects) and CD patients (6% underweight and 7.1% obese subjects). Underweight NCWS subjects were significantly younger and had a shorter clinical history than the overweight or obese ones. Unlike the other NCWS patients, none of them had a DQ2 and/or DQ8 haplotype. Overweight and obese NCWS patients were more frequently suffering from associated autoimmune diseases than the other BMI categories (*P* = 0.05). Compared to the CD controls, NCWS patients showed a higher frequency of Irritable Bowel Syndrome (IBS)-like (*P* = 0.01) and extraintestinal symptoms (*P* = 0.03) and a longer clinical history (*P* = 0.04), whereas weight loss was more frequent in CD (*P* = 0.02). Conclusions: NCWS patients showed a BMI distribution similar to CD patients. However, NCWS was found to be a heterogenous condition that regards BMI, and clinical characteristics differed between the underweight and overweight/obese patients.

## 1. Introduction

In recent years, a new gluten- or wheat-related disease has emerged, a condition labelled “non-celiac gluten sensitivity” (NCGS) or “non-celiac wheat sensitivity” (NCWS) [1,2]. It is very often a self-reported condition, since patients refer to intestinal and extra-intestinal symptoms caused by gluten and/or wheat ingestion, even though they do not have celiac disease (CD) or wheat allergy [1,3].

However, NCWS is a still largely undefined condition, much less widely known than CD, which has been historically considered the main gluten-related disease. There are, at present, no diagnostic markers for NCWS, its pathogenesis is uncertain, and it is debated whether NCWS patients really need to adhere to a strict gluten-free diet. CD is considered an autoimmune disease affecting both the small bowel and extra-intestinal sites and is determined by gluten ingestion in genetically predisposed persons who carry the Human Leukocyte Antigen (HLA)-DQ2 and/or -DQ8 haplotypes. By contrast, very few data are available about NCWS pathogenesis; previous studies have suggested a prevalent role for Fermentable Oligosaccharides-Disaccharides-Monosaccharides and Polyols (FODMAPs) [4] while others have hypothesized the activation of innate immunity [5] or a non-IgE-mediated food allergy [6].

Regarding diagnosis, patients with suspected CD can be evaluated by a combination of immunoassays, which offer a very high sensitivity, since if IgA anti-transglutaminase and/or IgG anti-Deamidate Gliadin Peptide assays are positive, then the test is considered positive for CD [7]. On the other hand, no biomarkers have been identified to date for NCWS.

Moreover, the clinical presentation, diarrhea, malabsorption, and malnutrition were formerly considered the classical symptoms of CD, due to the presence of intestinal villi atrophy [8]. Nevertheless, in the last few decades, the frequency of this typical clinical picture has progressively decreased, and atypical manifestations have increased [9]. Indeed, many reports indicate that CD can be found in normal or overweight subjects; hence, CD does not always present with malnutrition [10,11,12,13,14,15].

Finally, very few reports on NCWS have focused on nutritional status. In our experience, the “self-reported NCWS patients” condition includes both patients who report weight loss and thinness and others who report diffuse swelling, weight increase, and obesity, but all attribute their symptoms to eating gluten.

In this retrospective study, we reviewed our database of adult patients with a definitive diagnosis of NCWS based on a double-blind placebo-controlled wheat challenge (DBPCWC) method, to evaluate the proportion of patients who were underweight, overweight, or obese at diagnosis, and to search for possible correlations between their Body Mass Index (BMI) and other NCWS-related disease characteristics.

## 2. Patients and Methods

The clinical charts of the NCWS patients attending the outpatient clinics of the Department of Internal Medicine at the University Hospital of Palermo and the Department of Internal Medicine of the Hospital of Sciacca (both in southern Italy) were reviewed with a retrospective method. They had all been diagnosed with NCWS between January 2012 and March 2018. These charts included specific sections for the clinical presentation of the disease, physical examination, biochemical features, histological characteristics, and BMI. Incomplete clinical charts were excluded. A control group was also selected, composed of CD patients randomly chosen by a computer-generated method from subjects diagnosed during the same period, age- (±2 years) and sex-matched (±5%) with the NCWS patients.

### 2.1. Non-Celiac Wheat Sensitivity Diagnosis

CD or wheat allergy diagnoses had been ruled out in all the study patients, according to the following criteria:

(1) Negative serum assays for CD-anti-tissue transglutaminase (anti-tTG) IgA and anti-deamidated gliadin peptides (anti-DGP) IgG antibodies; (2) the absence of intestinal villous atrophy, documented in all the patients carrying the DQ2 and/or the DQ8 HLA haplotypes; (3) and negative IgE-mediated immune-allergy tests to wheat (skin prick tests and/or specific serum IgE detection). Furthermore, the criteria adopted in our patients, in accordance with our diagnostic protocol for NCWS diagnosis, were a resolution of the symptoms on a standard elimination diet without wheat, cow’s milk, yeast, and other food(s) causing self-reported symptoms, followed by symptom reappearance on a Double-Blind Placebo-Controlled Wheat Challenge (DBPCWC), performed as described previously [16,17]. As in previous studies, a double-blind placebo-controlled cow’s milk protein challenge and other open food challenges were also performed. Additional inclusion criteria were: age above 20 years, follow-up duration longer than 12 months after the initial diagnosis, and at least two outpatient visits during the follow-up period [16].

### 2.2. Celiac Disease Diagnosis

CD was diagnosed in the presence of positive serum anti-tTG and/or anti-DPG antibodies and duodenal villous atrophy at histologic examination, followed by the resolution of symptoms after commencement of the gluten-free diet. The above clinical investigations were always performed on a diet containing gluten (at least eight grams per day) followed for a minimum of 4 weeks [18].

### 2.3. Outcomes

BMI was used in our study to define the nutritional status of the patients. The World Health Organization (WHO) defines BMI < 18.5 as underweight, 18.5–24.9 as normal, 25.0–29.9 as overweight, and 30 or over as obese [19]. As these criteria are only applicable above the age of 20, younger patients were excluded. The BMI of the study patients was correlated with the following clinical and laboratory parameters: (1) gender; (2) age at diagnosis; (3) coexistent atopic diseases (i.e., respiratory allergy or atopic dermatitis); (4) coexistent autoimmune diseases; (5) the presence and kind of irritable bowel syndrome (IBS)-like symptoms (i.e., diarrhea, constipation, or both, occurring at least 6 months before diagnosis, and presenting during the previous 3 months); (6) the presence of functional dyspepsia, defined as postprandial fullness, early satiety, epigastric pain, and epigastric burning severe enough to interfere with usual activities and occurring at least 3 days per week over the previous 3 months, with an onset of at least 6 months beforehand [20]; (7) the presence of extraintestinal symptoms (i.e., a foggy brain, headache, fatigue, joint and muscle pain, leg or arm numbness); (8) the presence of weight loss; (9) symptom duration; and (10) the presence/absence of DQ2/DQ8 HLA haplotypes.

### 2.4. Statistical Analysis

Data were expressed as mean ± standard deviation when the distribution was Gaussian, and differences were calculated using Student’s *t* test. For variables with a non-Gaussian distribution, values were expressed as median and range and analyzed with the Mann-Whitney U test. To evaluate the correlation between the DQ2/DQ8 HLA haplotypes versus BMI score, the Spearman rank correlation test was used. The Chi-square test or Fisher‘s Exact test were used to compare categorical variables.

## 3. Results

Over the study period, we selected the clinical charts of 145 NCWS patients, another 40 having been excluded as they were incomplete; the NCWS study group, therefore, included 125 females and 20 males (mean age 37.1 ± 11.4 years). As a comparison, the clinical charts of 84 celiac patients (73 females, 11 males, mean age 39.8 ± 13.9 years) were evaluated.

### 3.1. BMI

**Figure 1:**
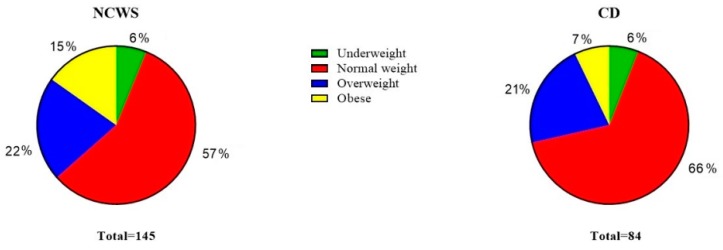
Distribution of underweight, normal weight, overweight and obese subjects in the non-celiac wheat sensitivity (NCWS) and celiac disease (CD) study patients.

Figure 1 shows the distribution of underweight, normal weight, overweight, and obese subjects in the NCWS and CD patients included in the study. Among the NCWS patients, nine were underweight (6.2%) and 22 obese (15.2%); the overall frequency of overweight plus obese NCWS patients was 37.9%. For the CD patients, five were underweight (6%) and six obese (7.1%); the overall percentage of overweight plus obese CD patients was 28.5%. There were no statistically significant differences between NCWS and CD patients for BMI distribution, even when overweight and obese patients were considered as a single subgroup.

### 3.2. Correlation of BMI with Clinical and Laboratory Parameters

Table 1 reports the correlations between BMI and the clinical and laboratory parameters in the NCWS patients. Underweight subjects were significantly younger at diagnosis than the overweight or obese subjects (underweight vs. overweight and obese, *P* = 0.04 and 0.03, respectively). Accordingly, symptom duration was longer in the overweight and obese patients than in the underweight patients (underweight vs. overweight and obese, *P* = 0.04 for both groups); the duration was also longer in the overweight subjects than in normal subjects (*P* = 0.04). All the underweight NCWS patients were negative for HLA DQ2 or DQ8 haplotypes, whereas these haplotypes were present in about half of the patients in the other BMI categories (HLA DQ2/DQ8 frequency: underweight vs. normal, overweight, and obese, *P*= 0.003, 0.005, and 0.07, respectively). Furthermore, coexistent autoimmune diseases were more frequent in the obese than in normal weight NCWS subjects (*P* = 0.05).

Table 2 compares the NCWS and CD patients in regard to the clinical variables evaluated in the study, as well as their HLA status. NCWS patients showed a higher frequency of IBS-like symptoms than CD subjects (*P* = 0.01), as an IBS-like clinical presentation was recorded in over 90% of cases; in particular, IBS-constipation was significantly more frequent in NCWS than in CD patients (*P* = 0.03). Furthermore, the frequency of extra-intestinal symptoms was higher in NCWS than in CD subjects (*P* = 0.03). On the other hand, weight loss as a presenting symptom was significantly more frequent in CD than in NCWS patients (*P* = 0.02). Symptoms duration was longer in NCWS than in CD patients (*P* = 0.04). Finally, as expected, the HLA haplotypes DQ2 and/or DQ8 were significantly more present in CD than in NCWS subjects (*P* < 0.001).

## 4. Discussion

To our knowledge, this is the first study aiming to evaluate BMI in NCWS patients and its correlation with other clinical and laboratory parameters, in comparison with a control group of celiac patients.

More than one-third of the whole NCWS group of patients were overweight/obese, whereas approximately 7% were underweight. Thus, in our study group, there was a high frequency of overweight/obese NCWS patients. This frequency, however, is similar (albeit slightly lower) to the percentage reported for the general Italian population; the Italian National Statistics Institute (ISTAT) reported a frequency of 35% for overweight and 10% for obese subjects in people living in Italy [21]. The same report indicated a lower frequency of underweight subjects (3%) than we observed in our NCWS group (7%) [21].

Multiple factors could influence the nutritional status of NCWS patients. One hypothesis is that, since NCWS patients (most of them self-reporting their wheat-related symptoms) tend to choose to consume processed gluten-free foods, they could be following a diet including high levels of lipids and sugars, and this, in turn, may put them at risk of becoming obese and developing metabolic syndrome [22,23,24]. On the other hand, it has been demonstrated that when altering their dietary habits, NCWS patients could experience a possible deficiency in macro and micronutrient intake due to dietary self-restrictions, such as lower mean amounts of carbohydrates, proteins, fiber, polyunsaturated fatty acids, calcium, and folates, (by avoiding fruit, vegetables, milk, and dairy products) when compared to a control population [25,26]. To support this, in a previous study we demonstrated a high frequency of osteopenia and osteoporosis in NCWS patients at diagnosis, which correlated with a tendency towards a low BMI [26].

The results of the present study seem to indicate that the clinical characteristics of the overweight/obese NCWS subjects and the underweight ones are different. Interestingly, overweight and obesity in NCWS patients were associated with a longer clinical history and a higher frequency of autoimmune diseases than in underweight subjects, respectively. In our study, the underweight NCWS patients were diagnosed before the overweight and obese ones. In addition, symptom duration in the overweight and obese NCWS patients was longer than in the underweight and normal ones. In other words, the lower the BMI, the sooner the NCWS diagnosis was made, and the shorter the symptom duration. A possible explanation could be that overweight and obese NCWS patients consult physicians later than underweight ones and are, therefore, also diagnosed later. Being underweight is perhaps self-perceived by patients as a more worrying clinical sign, pushing them to first consult a physician and/or undergo a diagnostic work-up, which finally leads to a diagnosis and therapy (i.e., a gluten-free diet). However, it cannot be ruled out that the clinical characteristics and BMI of the patients may independently change with aging and with the progression of the disease (i.e., the appearance of overweight and obese phenotypes). In addition, our data showed that overweight NCWS patients present a higher association with autoimmune diseases, particularly autoimmune thyroiditis, than normal weight subjects, and this disease is frequently complicated with hypothyroidism, which can increase BMI values if not diagnosed or correctly treated [27]. We also found a statistically significant difference between underweight NCWS patients and normal, overweight and obese ones in regards to the HLA haplotypes: the percentage of DQ2/DQ8 positive patients in the normal and overweight subgroups was in line (about 50%) with our and other authors’ previous data [1,16,28,29]. On the contrary, all the underweight patients were DQ2/DQ8 negative, suggesting the hypothesis that these patients have a different clinical condition and not a gluten-related disease. In particular, a coexistent eating disorder in this subgroup of patients cannot be ruled out.

Data from the celiac patients included in this study were in agreement with previous research, demonstrating the changes in the clinical presentation of the disease compared to the historical one (i.e., malabsorption syndrome), with an increasing rate of non-classical and subclinical phenotypes [9]. Accordingly, overweight and obesity are being increasingly recognized in CD, with several papers reporting from 8% to 40% of CD patients as overweight or even obese at diagnosis [10,11,12,13,14,15]. Our data were in this range, as we found 8% subjects obese and 21% overweight.

As expected, the comparison between the NCWS and CD groups showed a somewhat longer duration of clinical history in NCWS than in CD; this could be simply explained by the lack of a diagnostic biomarker for NCWS. However, the median duration of symptoms before reaching the NCWS and the CD diagnoses was far too long in both these diseases: the NCWS study patients had a median of about 10 years of symptoms before diagnosis, with a maximum of over 20 years. But it is noteworthy that the diagnostic delay was also long for CD, with a median of about 6 years (73 months); this should be unacceptable for a well-known clinical condition for which there are accurate serum markers.

Regarding the clinical presentation, there was a significantly higher frequency of IBS-like clinical presentations in NCWS than in CD patients. More than 90% of NCWS patients showed IBS-like symptoms, a result in agreement with many others in the literature, which underlines the overlap between IBS and NCWS [30]. Interestingly, we found a higher frequency of IBS-constipation in NCWS (20%) than in CD patients (8%). Previous studies have investigated the efficacy of the gluten-free/wheat-free diet in NCWS patients presenting with IBS-diarrhea [31,32], but our data seem to suggest that IBS-constipation could also benefit from a gluten-free diet. Our NCWS study patients also showed a higher frequency of extraintestinal symptoms (i.e., foggy mind, headache, fatigue, joint and muscle pain, leg or arm numbness) than the CD patients; 77% of the NCWS study patients showed extra-intestinal symptoms, and this could support the hypothesis that NCWS has an underlying immunologic condition, which can determine a multi-organ dysfunction [33,34]. Obviously, DQ2-DQ8 haplotypes were more frequently observed in CD patients than in NCWS patients. However, we confirmed that about 50% of NCWS patients had an HLA DQ2 and/or DQ8 haplotype, a frequency higher than in the general population, and in agreement with previous reports [16].

Some limitations of our study must be underlined. Firstly, this is a retrospective study and the data collection could be imprecise. Furthermore, we are unable to report other nutritional parameters apart from BMI (i.e., levels of hemoglobin concentration, ferritin, albumin, vitamin B12, and serum folate). We have no data on patients’ dietary habits, and our considerations about the factors associated with underweight and overweight/obesity must be considered speculative. In the case of the underweight NCWS patients, eating disorders were not regularly excluded by means of psychometric methods.

However, our study is the first to focus on BMI in NCWS patients and to attempt to correlate the nutritional status of patients with the other clinical characteristics of this disease. Furthermore, the major strength of the study is the patient selection process, based on an NCWS diagnosis made by a rigorous DBPCWC protocol, over a six-year period.

In conclusion, our study suggests that, on the basis of our BMI findings, NCWS patients belong to different populations, in keeping with the well-known heterogeneity of this condition. A quite large subgroup of NCWS patients were overweight or obese and showed a longer clinical history and a higher frequency of associated autoimmune diseases, especially autoimmune thyroiditis. A smaller subgroup, including underweight subjects, had a shorter clinical history and was apparently characterized by a lack of the DQ2/DQ8 haplotypes.

## Figures and Tables

**Table 1:** Correlations between Body Mass Index (BMI) and clinical and laboratory variables in 145 NCWS patients. Values are given as an absolute number and as a percentage (in brackets) in the respective BMI subgroups.

	BMI Score			
	Underweight (Group 1, *n* = 9)	Normal Weight (Group 2, *n* = 82)	Overweight (Group 3, *n* = 32)	Obese (Group 4, *n* = 22)
Sex (Female/Male)	7/2	70/12	29/3	19/3
Age	30.4 ± 10.3	35.6 ± 12	39.7 ± 9.5	40.2 ± 10.8
Atopic diseases	4 (44%)	34 (41%)	12 (38%)	8 (36%)
Autoimmune diseases	2 (22%)	13 (16%)	6 (19%)	9 (41%)
IBS-like presentation	8 (89%)	75 (92%)	32 (100%)	20 (91%)
Diarrhea	4 (44%)	43 (52%)	17 (53%)	10 (45%)
Constipation	1 (11%)	16 (20%)	8 (25%)	4 (18%)
Alternate bowel movements	3 (33%)	16 (19%)	5 (15%)	6 (27%)
Functional dyspepsia	4 (44%)	40 (51%)	18 (56%)	13 (59%)
Extraintestinal symptoms	7 (78%)	61 (74%)	27 (44%)	17 (77%)
Weight loss	4 (44%)	17 (21%)	6 (19%)	3 (14%)
Symptoms duration (median and range in months)	96 (27–120)	92 (24–180)	125 (45–264)	120 (24–288)
DQ2/DQ8 HLA haplotypes				
DQ2and DQ8				
negative	9 (100%)	37 (45%)	15 (48%)	16 (73%)
DQ2-positive	0	29 (36%)	11 (35%)	4 (18%)
DQ8-positive	0	14 (17%)	6 (17%)	2 (9%)
DQ2/DQ8 positive	0	2 (2%)	0	0

**Table 2:** Comparison of clinical variables frequency and HLA status in 145 patients suffering from Non-Celiac Wheat Sensitivity (NCWS) and in 84 sex- and age-matched Celiac Disease (CD) patients, chosen as controls.

	NCWS (*n* = 145)	CD (*n* = 84)	*P* Value
Atopic diseases	58 (40%)	33 (39%)	ns
Autoimmune diseases	30 (21%)	24 (28%)	ns
IBS-like presentation	135 (93%)	65 (77%)	0.01
Diarrhea	74 (51%)	34 (40%)	ns
Constipation	29 (20%)	7 (8%)	0.03
Alternate bowel movements	30 (21%)	24 (28%)	ns
Functional dyspepsia	75 (52%)	45 (53%)	ns
Extraintestinal symptoms	112 (77%)	52 (62%)	0.03
Weight loss	30 (21%)	33 (39%)	0.02
Symptoms duration (median and range in months)	120 (24–288)	73 (15–228)	0.04
BMI score			
Underweight	9 (6%)	5 (6%)	ns
Normal weight	82 (57%)	55 (65%)	ns
Overweight	32 (22%)	18 (21%)	ns
Obese	22 (15%)	6 (8%)	ns
DQ2/DQ8 HLA			
haplotypes			
DQ2/DQ8 negative	77 (53%)	0	
DQ2-positive	44 (30%)	72 (86%)	
DQ8-positive	22 (15%)	8 (9%)	<0.001
DQ2/DQ8 positive	2 (2%)	4 (5%)	
